# The association of asthma duration with body mass index and Weight-Adjusted-Waist index in a nationwide study of the U.S. adults

**DOI:** 10.1186/s40001-023-01089-4

**Published:** 2023-03-14

**Authors:** Xiaoxiao Han, Xiaofang He, Gui Hao, Lifang Cao, Yinliang Qi, Kexing Han

**Affiliations:** 1 Department of Hyperbaric Oxygen, The Second People’s Hospital of Hefei, Hefei Hospital Affiliated to Anhui Medical University, Hefei, Anhui China; 2 Department of Neurology and Clinical Research Center of Neurological Disease, The Second Affiliated Hospital of Suzhou University, Suzhou, Jiangsu China; 3 Bozhou People’s Hospital Affiliated with Anhui Medical University, Hefei, Anhui China; 4 The First Affiliated Hospital of Anhui Medical University, No. 218 Jixi Road, Shushan District, Hefei, Anhui Province People’s Republic of China

**Keywords:** Asthma, Duration of asthma, Obesity, Body mass index, Weight-Adjusted-Waist index, Cross-sectional study, NHANES

## Abstract

**Backgrounds:**

The association between obesity and asthma has been of interest, but whether the duration of asthma has an effect on obesity is still limitedly studied.

**Aim:**

The purpose of this study was to investigate the association between asthma duration and obesity-related indexes, where obesity-related indexes include Body mass index (BMI) and Weight-adjusted-waist index (WWI).

**Methods:**

Data from National Health and Nutrition Examination Survey (NHANES) 2009–2018 were obtained to conduct this cross-sectional study. Duration of asthma was used as the independent variable and obesity-related indexes as the response variables. Multiple linear regression was used to assess the association between the independent variable and the response variables, and subsequently smoothed curve fitting and threshold effect analysis were performed to clarify whether there was a nonlinear correlation between the independent variable and the response variables. Finally, subgroup analysis was conducted to find sensitive populations.

**Results:**

A total of 9170 participants were included in the analysis. Asthma duration was statistically different between the two groups when all participants were grouped by median WWI (Q1 < 11.65, Q2 ≥ 11.65) (*P* < 0.001), but not by median BMI (Q1 < 31.8, Q2 ≥ 31.8) (*P* = 0.130). There was a positive association between asthma duration and WWI [*β* = 0.016, 95% CI (0.016, 0.017)], but a negative one with BMI [*β* = − 0.098, 95% CI (− 0.112, − 0.085)], and the correlations between the independent and response variables became more pronounced with increasing asthma duration (*P* for trend < 0.01). In addition, there were nonlinear relationships between asthma duration with BMI and WWI (log likelihood ratio < 0.001), with the best valid inflection points for asthma duration being 2 years (with WWI as the response variable) and 3 years (with BMI as the response variable), respectively. In the subgroup analysis, the positive association between asthma duration and WWI was more pronounced in the participants who were male, aged less than 40 years, and had asthma onset before 12 years of age. In contrast, when BMI was used as the response variable, the negative association between it and asthma duration was more pronounced among participants of female, aged 60 years or older, and with asthma onset less than 12 years of age.

**Conclusions:**

In US adults, asthma duration might cause changes in obesity-related indexes. Longer asthma duration might cause weight loss, but might increase the risk of abdominal obesity.

## Introduction

Asthma is a common chronic disease in the respiratory system, but the prevention and control of asthma is complicated by its numerous causes and the absence of a single biomarker that can be identified in time [1]. As the understanding of asthma has gradually increased, the association between obesity and asthma has attracted the interest of researchers [2, 3]. Although a study showed no significant positive association between obesity and the risk of readmission in asthma patients [4], most researchers have concluded that obesity increases the risk of suffering from asthma [5–8], especially since previous studies recommended 10 traits (including obesity) which could predict future asthma attacks and demonstrated a 13% increase in the risk of asthma attacks with each additional trait [9, 10]. In addition, some studies also indicated that obesity was strongly associated with more severe asthma and poorer lung function [11, 12].

In contrast to previous views, researchers have suggested a bidirectional correlation between obesity and asthma [13]. In addition to the presence or absence of asthma, the duration of asthma is a notable presence in the management of asthma. Previous studies demonstrated that although asthma duration would not affect the severity of asthma [14], it could influence eventual pulmonary function, such as higher diffusing capacity and lung hyperinflation [15]. In addition, it has been suggested that asthma duration of less than 10 years might be a beneficial factor in the therapeutic effect of patients to IL-5 biologics [16]. It could be shown that, in addition to the presence or absence of asthma, the duration of asthma has a subtle effect on the organism. However, whether asthma duration could increase the burden of obesity remains rarely reported. In a limited number of small sample cross-sectional studies, Holguin F reported a positive association between asthma duration and BMI in patients with early-onset asthma (< 12 years) [17], but Ahmadiafshar A did not reach similar conclusions in their study [18]. Therefore, whether asthma duration would cause changes in obesity-related indexes is still not precisely described.

Previous studies have used body mass index (BMI) as a measure of obesity [19, 20], but the accuracy of BMI has been called into question in recent years [21, 22]. More researchers believe that BMI is more appropriate as a crude estimate of obesity [23]. Asthma is characterized by the presence of persistent chronic airway inflammation, the vast majority of asthmatic patients experience the use of glucocorticoids [1], and the most typical side effect of steroid medications is the development of central obesity [24]. Previous study demonstrated that body fat had a negative effect on human pulmonary function and that this negative effect was inevitable for non-obese populations [25]. As researchers have gained a better understanding of obesity, recent studies have shown that central obesity responds to a more realistic body fat situation [26] and that central (or androgenic) obesity, which is primarily a response to visceral fat distribution, was associated with asthma and impaired pulmonary function in adolescents and adults compared to peripheral obesity [27]. To better reflect the true situation of obesity, some researchers first proposed a new obesity index and named it the weight-adjusted waist index (WWI) [28]. Due to the adjustment for body weight, in the adult population this index mainly reflects weight-independent central obesity [28] and has been shown by many researchers to have better accuracy compared to BMI [29–32]. However, the association between asthma duration and WWI has still not been reported.

Therefore, the aim of this study was to investigate the association between asthma duration and obesity-related indexes with data from the National Health and Nutrition Examination Survey (NHANES), where obesity-related indexes include BMI, an indicator of traditional evaluation of obesity, and WWI, an indicator of central obesity.

## Methods

### Data source

The National Health and Nutrition Examination Survey (NHANES), established in the 1990s, is a public service survey of the entire United States population in which demographic, dietary, physical, lifestyle, medical, and laboratory information is regularly collected to assess the health and nutritional status of the nation's population. The data in NHANES are currently updated every 2 years, and additional information may be added each time the data is updated. The NHANES survey protocol was approved by the National Center for Health Statistics (NCHS) Ethics Review Board, and written informed consent was provided to all participants. Because the NHANES data are open to the public, ethical review of this study was exempt.

### Participants

**Fig. 1:**
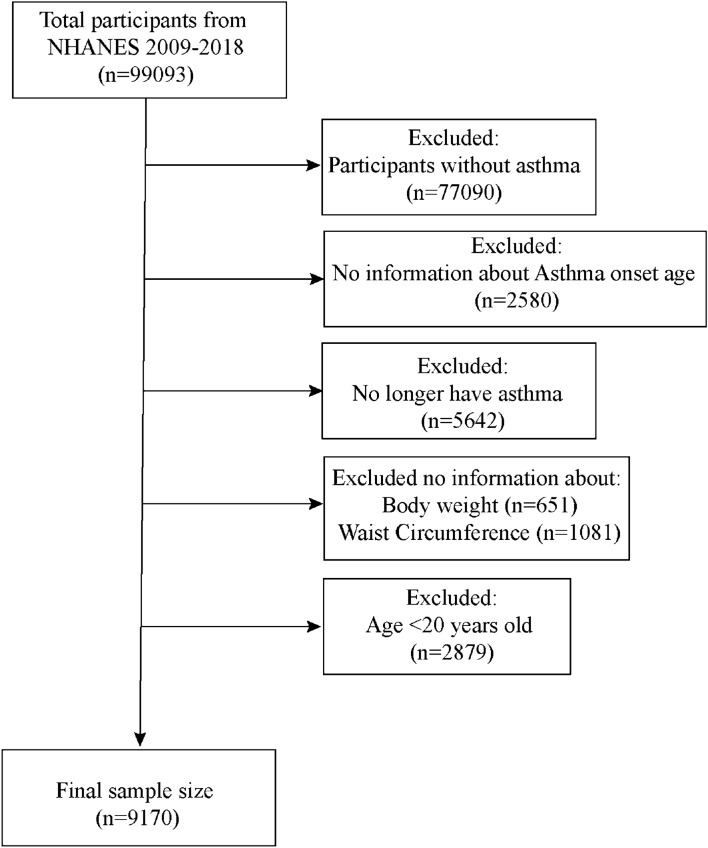
Flow chart for participants

NHANES 2009–2018 included complete information on questionnaires related to asthma and information on covariates that needed to be adjusted for in the follow-up models was also complete. A total of 99,093 participants enrolled in NHANES 2009–2018, and we first excluded 77,090 participants who did not have asthma. Subsequently, we removed 2580 participants without clear information on age of asthma onset. Among the 19,423 participants with clear information on the age of asthma onset, we removed 5642 participants who no longer had asthma. At the same time, we removed 651 participants with missing weight information and 1081 participants with missing waist circumference information. Finally, we removed 2879 participants who were younger than 20 years of age. Finally, a total of 9170 participants were included in this study (Fig. 1).

### Definition of asthma duration

Information about the participants’ asthma status was obtained from a medical conditions questionnaire. Firstly, participants who responded positively to the question “Ever been told you have asthma?” were retained. Subsequently, participants who responded positively to the question “Still have asthma?” was reserved. Finally, participants that were able to give an exact answer to the question “Age when first had asthma?” were included in this study. Overall, asthma duration = age (years)−age at onset of asthma (years) and all enrolled participants in the study were still currently suffering from asthma.

### BMI and WWI

BMI and WWI were obtained from body measurement information in the physical examination module, and body measurements were collected by trained health technicians at mobile examination centers (MECs). BMI was calculated as weight in kilograms divided by the square of height in meters, and WWI was calculated as waist circumference in centimeters divided by the square root of weight in kilograms [33]. Body measurement information from NHANES 2009–2018 were subjected to review and the measurement methods remained consistent over this time period.

### Covariates

To better estimate the association between asthma duration and obesity-related indices, we included a number of factors with potential effects on asthma duration and/or obesity-related indices as covariates in the subsequent models, based on previous studies [17, 18, 34, 35]. Demographic information included participants' age, race (black, white, and other races), education level (less than high school, high school, and more than high school), and ratio of family income to poverty (PIR). Dietary information was extracted from a 24-h dietary questionnaire, which included participant self-reported information on total nutrients, and the mean of the sum of the participant-specific nutrient intakes for the 1st and 2nd 24-h periods was ultimately included in this study. Dietary information included in this study included energy (kcal), sugar (gm), fat (gm), cholesterol (mg), and moisture (gm). According to NHANES official instructions, the “moisture” included in this study refers not only to drinking water, but to all moisture in the diet, including food and beverages. Participants who answered yes to the question “Smoked at least 100 cigarettes in life?” were identified as smokers and were classified according to their current smoking status (now, ever, never) with their responses to the question “Do you now smoke cigarettes?”. Alcohol consumption was defined as “more than 12 drinks in the past 1 year”. Diabetes and hypertension were defined by the participants’ responses to the questions “Doctor told you have diabetes?” and “Ever told you had high blood pressure?”. Due to limitations of the NHANES data, we were unable to capture the specific types of medications used by participants, we obtained information on the participants’ prescription medication use, and a positive response to the question “Taken prescription medicine past month?” was identified as prescription medication use. A “prescription medication” in this context would be any prescription medication used by the participant within the past month.

### Statistical analysis

Analysis of all data was completed in R (http://www.R-project.org) and EmpowerStats (http://www.empowerstats.com). Because both independent and response variables were continuous variables in this study, we grouped participants based on median WWI and BMI as WWI_Q1_ (< 11.65), WWI_Q2_ (≥ 11.65), BMI_Q1_ (< 31.8), and BMI_Q2_ (≥ 31.8), respectively. For comparisons between groups of information on general characteristics of participants, continuous variables were expressed as mean ± standard deviation, and rates or percentages were used to describe categorical variables. Since covariates might have missing values, the missing values of continuous variables were interpolated using the mean of the variable if the percentage of missing values was less than 10% of the total sample size, otherwise we grouped the continuous variables according to the interquartile distribution and set the missing values as “Unclear”. The missing values of the categorical variables were grouped separately and named “Unclear”. The association between asthma duration with WWI and BMI was assessed separately using multiple linear regression with asthma duration as the independent variable. We used β to respond the effect values between the independent variable and the respondent variable, which means that for each unit increase in the independent variable the respondent variable will increase by β unit values, and we also calculated the 95% confidence interval (95% CI) for each effect value. Three models were generated by adjusting for different covariates, Model 1 (no covariates adjusted), Model 2 (age, gender, race, and asthma onset age were adjusted), and Model 3 (all covariates were adjusted). Participants were divided into four groups based on the quartile distribution of asthma duration to assess whether there was a trend change (*P* for trend) in the correlations between the independent variable and obesity-related indexes (WWI and BMI) as the asthma duration increased. Smoothing curves were used to assess whether there were non-linear relationships between the effects of asthma duration and obesity indexes, and threshold effect analysis was used to determine the best valid inflection points for the independent variable. A log-likelihood ratio (LLR) of less than 0.05 was used in the threshold effects analysis as the basis for adopting the nonlinear model. Subsequently, a subgroup analysis of the association between the independent variable and the response variables was performed to find sensitive individuals.

## Results

### Characteristics of participants

A total of 9170 participants were eventually enrolled in this study, of whom 3,121 were male and 6049 were female. The mean age of all participants was 56.43 ± 15.51 years with a mean duration of asthma was 24.84 ± 18.78 years. The mean WWI was 11.61 ± 0.83 and BMI was 33.19 ± 8.78 kg/m^2^.

When grouped by median WWI (11.65), asthma duration was statistically different between the two groups (*P* < 0.001). However, when grouped by median BMI (31.8 kg/m^2^), no significant statistical difference in asthma duration was seen between the two groups (*P* = 0.130). In addition, there were statistically significant differences in age, gender, and asthma onset age of participants between groups regardless of the median obesity index used for grouping (*P* < 0.001). The results of the baseline characteristics of the participants were displayed in Tables 1 and 2.

**Table 1:** Characteristics of participants grouped by median WWI

Characteristics	WWI_Q1_(*n* = 4589)	WWI_Q2_(*n* = 4581)	*P*-value
Gender (%)			< 0.001
Male	40.84	27.22	
Female	59.16	72.78	
Age (years)	52.62 ± 16.26	60.24 ± 13.69	< 0.001
Stratified by age (years) (%)			< 0.001
20–39	24.43	8.12	
40–59	37.96	32.90	
60–80	37.61	58.98	
Asthma onset age (years)	23.89 ± 18.00	25.79 ± 19.48	< 0.001
Stratified by asthma onset age (years) (%)			< 0.001
< 12	29.92	21.48	
≥ 12	70.08	78.52	
Asthma duration (years)	23.89 ± 18.00	25.79 ± 19.48	< 0.001
Quartiles of asthma duration (years) (%)			< 0.001
Q1 (0–9)	25.02	24.10	
Q2 (10–19)	22.79	22.75	
Q3 (20–38)	28.50	25.63	
Q4 (39–79)	23.69	27.53	
Race (%)			< 0.001
White	46.55	48.66	
Black	28.94	19.91	
Other races	24.52	31.43	
Education level (%)			< 0.001
< High school	22.99	29.71	
High school	24.06	23.34	
> High school	52.91	46.82	
Unclear	0.04	0.13	
PIR	2.19 ± 1.53	1.87 ± 1.35	< 0.001
Stratified by PIR (%)			< 0.001
< 1.35	40.55	44.97	
1.35–3.45	36.33	39.75	
> 3.45	23.12	15.28	
Smoking (%)			0.532
Now	13.88	13.67	
Ever	25.04	25.15	
Never	40.01	38.94	
Unclear	21.07	22.24	
Alcohol (%)			< 0.001
Yes	63.04	56.01	
No	29.81	37.81	
Unclear	7.15	6.18	
Physical activity intensity (%)			0.187
Light	26.32	27.31	
Moderate	22.75	23.99	
Vigorous	33.34	31.59	
Unclear	17.59	17.11	
Hypertension (%)			< 0.001
Yes	56.35	74.50	
No	43.65	25.13	
Unclear	0.00	0.37	
Diabetes (%)			< 0.001
Yes	21.86	49.79	
Borderline	5.95	3.21	
No	72.11	46.82	
Unclear	0.09	0.17	
Prescription medications (%)			0.830
Yes	71.30	71.47	
No	28.68	28.49	
Unclear	0.02	0.04	
Energy (kcal) (%)			< 0.001
3.11–1565.4	25.23	31.26	
1565.5–2043.4	26.28	30.30	
2043.5–7878.5	31.49	25.13	
Unclear	17.00	13.32	
Sugars (gm) (%)			< 0.001
3.79–69.64	25.56	31.02	
69.65–113.98	26.59	29.95	
113.99–688.79	30.86	25.71	
Unclear	17.00	13.32	
Fat (gm) (%)			< 0.001
3.88–54.26	25.74	30.84	
54.40–80.77	27.85	28.51	
80.85–279.40	29.42	27.33	
Unclear	17.00	13.32	
Cholesterol (mg)			< 0.001
0–164.5	28.44	27.94	
165.0–310.5	24.80	31.89	
311.0–1692.0	29.77	26.85	
Unclear	17.00	13.32	
Moisture (gm) (%)			< 0.001
418.80–2127.31	28.50	28.03	
2127.32–3130.05	28.05	28.33	
3130.06–11,897.20	26.45	30.32	
Unclear	17.00	13.32	

Mean ± SD for continuous variables: *P*-value was calculated by weighted linear regression model

% for Categorical variables: *P*-value as calculated by weighted chi-square test

**Table 2:** Characteristics of participants grouped by median BMI (kg/m^2^)

Characteristics	BMI_Q1_(n = 4560)	BMI_Q2_(n = 4610)	P-value
Gender (%)			< 0.001
Male	39.91	28.22	
Female	60.09	71.78	
Age (years)	57.53 ± 16.67	55.34 ± 14.19	< 0.001
Stratified by age (years) (%)			< 0.001
20–39	17.57	15.01	
40–59	30.09	40.72	
60–80	52.35	44.27	
Asthma onset age (years)	32.83 ± 23.04	30.36 ± 20.69	< 0.001
Stratified by asthma onset age (years) (%)			0.705
< 12	25.88	25.53	
≥ 12	74.12	74.47	
Asthma duration (years)	24.70 ± 19.12	24.97 ± 18.43	0.130
Quartiles of asthma duration (years) (%)			0.211
Q1 (0–9)	24.76	24.36	
Q2 (10–19)	23.25	22.30	
Q3 (20–38)	26.10	28.03	
Q4 (39–79)	25.90	25.31	
Race (%)			< 0.001
White	50.86	44.38	
Black	20.55	28.26	
Other races	28.60	27.35	
Education level (%)			0.003
< High school	28.05	24.66	
High school	22.98	24.40	
> High school	48.90	50.82	
Unclear	0.07	0.11	
PIR	2.19 ± 1.51	1.87 ± 1.37	< 0.001
Stratified by PIR (%)			< 0.001
< 1.35	38.97	46.51	
1.35–3.45	38.33	37.74	
> 3.45	22.70	15.75	
Smoking (%)			< 0.001
Now	12.83	14.71	
Ever	27.30	22.91	
Never	40.37	38.59	
Unclear	19.50	23.80	
Alcohol (%)			< 0.001
Yes	62.04	57.05	
No	30.70	36.88	
Unclear	7.26	6.07	
Physical activity intensity (%)			0.107
Light	27.46	26.18	
Moderate	22.50	24.23	
Vigorous	33.07	31.87	
Unclear	16.97	17.72	
Hypertension (%)			< 0.001
Yes	56.78	73.97	
No	43.03	25.86	
Unclear	0.20	0.17	
Diabetes (%)			< 0.001
Yes	23.40	48.09	
Borderline	72.48	46.62	
No	4.12	5.03	
Unclear	0.00	0.26	
Prescription medications (%)			0.026
Yes	72.52	70.26	
No	27.43	29.72	
Unclear	0.04	0.02	
Energy (kcal) (%)			< 0.001
3.11–1565.4	28.77	27.72	
1565.5–2043.4	26.62	29.93	
2043.5–7878.5	26.75	29.85	
Unclear	17.85	12.49	
Sugars (gm) (%)			< 0.001
3.79–69.64	29.50	27.09	
69.65–113.98	27.06	29.46	
113.99–688.79	25.59	30.95	
Unclear	17.85	12.49	
Fat (gm) (%)			< 0.001
3.88–54.26	28.05	28.52	
54.40–80.77	28.07	28.29	
80.85–279.40	26.03	30.69	
Unclear	17.85	12.49	
Cholesterol (mg) (%)			< 0.001
0–164.5	29.47	26.92	
165.0–310.5	26.10	30.56	
311.0–1692.0	26.58	30.02	
Unclear	17.85	12.49	
Moisture (gm) (%)			< 0.001
418.80–2127.31	31.10	25.47	
2127.32–3130.05	26.71	29.65	
3130.06–11,897.20	24.34	32.39	
Unclear	17.85	12.49	

Mean ± SD for continuous variables: *P*-value was calculated by weighted linear regression model

% for Categorical variables: *P*-value as calculated by weighted chi-square test

### The association between the asthma duration and obesity indexes

**Fig. 2:**
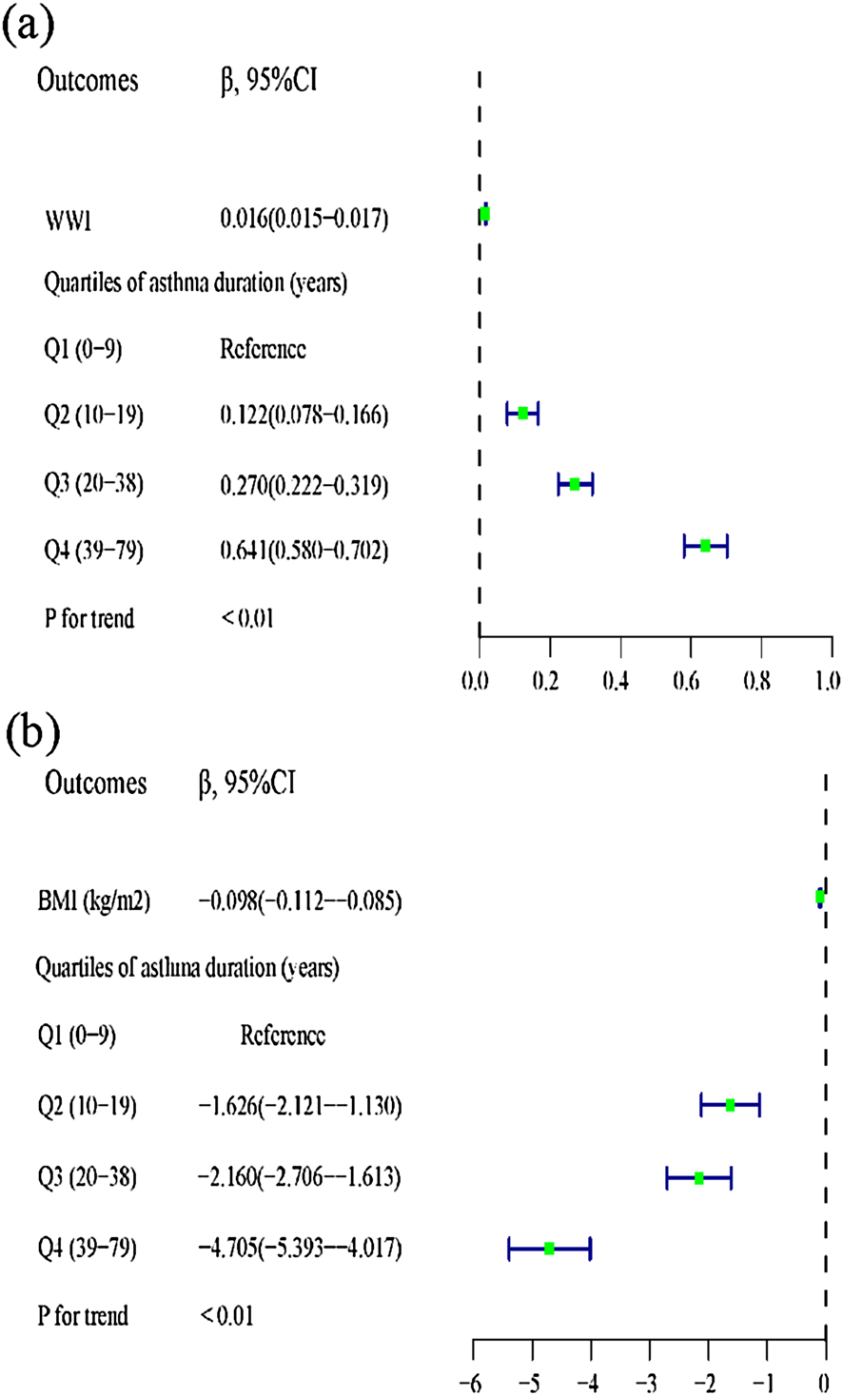
Trend test for the association between asthma duration and obesity-related indexes. **a** Trend test for the association between asthma duration and WWI. **b** Trend test for the association between asthma duration and BMI. *The green squares represents the effect value. The blue line represents the 95% confidence interval of the effect value. *All the covariates were adjusted

**Fig. 3:**
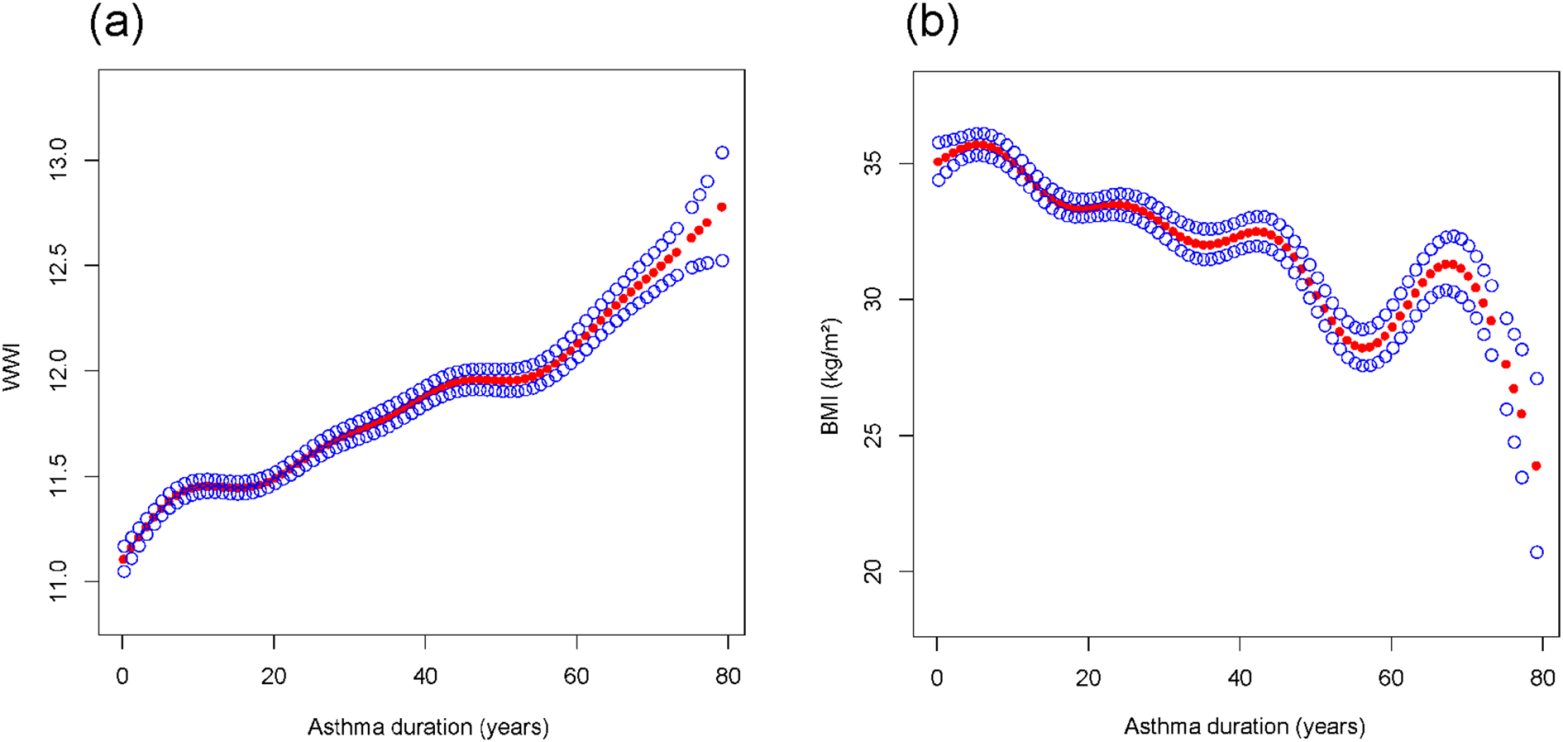
The association between the asthma duration and obesity indexes. **a** The association between the asthma duration and WWI. **b** The association between the asthma duration and BMI. *Solid rad line represents the smooth curve fit between variables. Blue bands represent the 95% of confidence interval from the fit. *All the covariates were adjusted

In the all-adjusted model, there was a positive association between asthma duration and WWI [*β* = 0.016, 95% CI (0.015, 0.017)]. Interestingly, the relationship between asthma duration and BMI was negative [*β* = − 0.098, 95% CI (− 0.112, − 0.085)]. Furthermore, the correlations between asthma duration and both obesity indexes became more significant with increasing asthma duration (*P* for trend < 0.01) (Table 3, Fig. 2). A smoothed curve fitting analysis for the association between asthma duration and these two obesity-related indexes revealed a significant nonlinear correlation between the independent and the responding variables (Fig. 3). Subsequently, threshold effect analysis suggested that the positive correlation between asthma duration and WWI switched from [*β* = 0.09, 95% CI (0.05, 0.14)] to [*β* = 0.02, 95% CI (0.01, 0.02)] when asthma duration exceeded 2 years. And when the response variable was BMI, threshold effect analysis indicated a positive association between asthma duration and BMI when asthma duration did not exceed 3 years [*β* = 0.41, 95% CI (0.11, 0.71)]. However, once the duration of asthma exceeded 3 years, the relationship between asthma duration and BMI turned negative [*β* = − 0.10, 95% CI (− 0.12, − 0.09)] (Table 4).

**Table 3:** The association of asthma duration with WWI and BMI (kg/m^2^)

Outcomes	Model 1 *β*, (95% CI)	Model 2 *β*, (95% CI)	Model 3 *β*, (95% CI)
WWI	0.003 (0.002, 0.004)	0.021 (0.019, 0.022)	0.016 (0.015, 0.017)
Quartiles of asthma duration (years)			
Q1 (0–9)	Reference	Reference	Reference
Q2 (10–19)	− 0.048 (− 0.097, 0.001)	0.157 (0.110, 0.204)	0.122 (0.078, 0.166)
Q3 (20–38)	− 0.126 (− 0.173, − 0.079)	0.337 (0.286, 0.388)	0.270 (0.222, 0.319)
Q4 (39–79)	0.125 (0.077, 0.172)	0.868 (0.808, 0.928)	0.641 (0.580, 0.702)
P for trend	< 0.01	< 0.01	< 0.01
BMI (kg/m^2^)	− 0.006 (− 0.016, 0.003)	− 0.031 (− 0.045, − 0.018)	− 0.098 (− 0.112, − 0.085)
Quartiles of asthma duration (years)			
Q1 (0–9)	Reference	Reference	Reference
Q2 (10–19)	− 0.994 (− 1.516, − 0.472)	− 1.252 (− 1.786, − 0.719)	− 1.626 (− 2.121, − 1.130)
Q3 (20–38)	− 0.434 (− 0.934, 0.067) 0.08929	− 1.315 (− 1.896, − 0.735)	− 2.160 (− 2.706, − 1.613)
Q4 (39–79)	− 0.501 (− 1.008, 0.006)	− 1.794 (− 2.482, − 1.106)	− 4.705 (− 5.393, − 4.017)
*P* for trend	< 0.01	< 0.01	< 0.01

Model 1 = no covariates were adjusted. Model 2 = Model 1 + gender, race were adjusted. Model 3 = Model 2 + asthma onset age, education level, PIR, smoking, alcohol, physical activity intensity, hypertension, diabetes, prescription medications, energy, sugars, fat, cholesterol, moisture were adjusted

**Table 4:** Threshold effect analysis for the association of asthma duration with BMI (kg/m^2^) and WWI

Outcomes	BMI (kg/m^2^)	WWI
Linear effect model		
β, (95%CI)	− 0.10 (− 0.11, − 0.08)	0.02 (0.01, 0.02)
Non-linear model		
Inflection point (K)	3	2
β, (95%CI) (< K)	0.41 (0.11, 0.71)	0.09 (0.05, 0.14)
β, (95%CI) (≥ K)	− 0.10 (− 0.12, − 0.09)	0.02 (0.01, 0.02)
LLR	< 0.001	< 0.001

^*^Gender, race, asthma onset age, education level, PIR, smoking, alcohol, physical activity intensity, hypertension, diabetes, prescription medications, energy, sugars, fat, cholesterol, moisture were adjusted in the models. LLR: Log-likelihood ratio; K: Inflection point.

### Results of subgroup analysis

In order to identify the stability of the association between asthma duration and the two obesity indexes in this study and to find sensitive cohorts, we performed a subgroup analysis. When WWI was the dependent variable, the positive association between asthma duration and WWI was more pronounced among male [*β* = 0.022, 95% CI (0.020, 0.024)], aged less than 40 years [*β* = 0.021, 95% CI (0.013, 0.029)], and asthma onset at age less than 12 years [*β* = 0.017, 95% CI (0.015, 0.019)] (Table 5). When BMI was taken as the response variable, the negative association between asthma duration and BMI was more pronounced in participants who were female [β = − 0.106, 95% CI (− 0.124, − 0.087)], aged 60 years or older [*β* = − 0.268, 95% CI (− 0.305, − 0.232)], and with asthma onset younger than 12 years [*β* = − 0.070, 95% CI (− 0.092, − 0.048)] (Table 6).

**Table 5:** A subgroup analysis of the association between asthma duration and WWI

Characteristics	Model 1 β, (95% CI)	Model 2 β, (95% CI)	Model 3 β, (95% CI)
Gender			
Male	0.002 (0.001, 0.004)	0.026 (0.024, 0.028)	0.022 (0.020, 0.024)
Female	0.004 (0.003, 0.005)	0.018 (0.016, 0.019)	0.014 (0.012, 0.015)
Stratified by age (years)			
20–39	0.002 (− 0.003, 0.007)	0.037 (0.030, 0.045)	0.021 (0.013, 0.029)
40–59	− 0.004 (− 0.006, − 0.002)	0.012 (0.008, 0.017)	− 0.001 (− 0.006, 0.003)
60–80	0.002 (0.001, 0.003)	0.014 (0.011, 0.017)	0.016 (0.013, 0.019)
Race			
White	0.005 (0.004, 0.006)	0.023 (0.021, 0.025)	0.018 (0.016, 0.019)
Black	0.000 (− 0.002, 0.002)	0.012 (0.010, 0.015)	0.003 (0.000, 0.006)
Other races	0.005 (0.003, 0.006)	0.024 (0.022, 0.026)	0.020 (0.018, 0.022)
Stratified by asthma onset age (years)			
< 12	0.018 (0.010, 0.027)	0.018 (0.010, 0.026)	0.021 (0.013, 0.029)
12–39	− 0.002 (− 0.005, 0.002)	− 0.001 (− 0.004, 0.002)	− 0.001 (− 0.004, 0.002)
≥ 40	0.005 (0.002, 0.009)	0.007 (0.004, 0.010)	0.009 (0.005, 0.012)

**Table 6:** A subgroup analysis of the association between asthma duration and BMI (kg/m^2^)

Characteristics	Model 1 β, (95% CI)	Model 2 β, (95% CI)	Model 3 β, (95% CI)
Gender			
Male	− 0.001 (− 0.014, 0.012)	− 0.012 (− 0.032, 0.008)	− 0.077 (− 0.097, − 0.058)
Female	− 0.006 (− 0.019, 0.007)	− 0.043 (− 0.061, − 0.025)	− 0.106 (− 0.124, − 0.087)
Stratified by age (years)			
20–39	0.064 (0.012, 0.117)	0.313 (0.225, 0.400)	0.136 (0.054, 0.219)
40–59	− 0.055 (− 0.073, − 0.036)	− 0.069 (− 0.125, − 0.014)	− 0.202 (− 0.255, − 0.150)
60–80	0.014 (0.003, 0.025)	− 0.289 (− 0.326, − 0.251)	− 0.268 (− 0.305, − 0.232)
Race			
White	− 0.017 (− 0.031, − 0.004)	− 0.031 (− 0.050, − 0.011)	− 0.108 (− 0.127, − 0.089)
Black	− 0.010 (− 0.030, 0.009)	− 0.029 (− 0.058, − 0.001)	− 0.120 (− 0.151, − 0.090)
Other races	0.010 (− 0.008, 0.028)	− 0.043 (− 0.070, − 0.017)	− 0.118 (− 0.146, − 0.090)
Stratified by asthma onset age (years)			
< 12	− 0.096 (− 0.134, − 0.058)	− 0.076 (− 0.114, − 0.038)	− 0.071 (− 0.106, − 0.035)
12–39	0.024 (− 0.011, 0.059)	0.018 (− 0.016, 0.052)	0.026 (− 0.007, 0.059)
≥ 40	0.046 (− 0.055, 0.148)	0.034 (− 0.066, 0.133)	0.016 (− 0.076, 0.108)

Model 1 = no covariates were adjusted. Model 2 = Model 1 + gender, race were adjusted. Model 3 = Model 2 + asthma onset age, education level, PIR, smoking, alcohol, physical activity intensity, hypertension, diabetes, prescription medications, energy, sugars, fat, cholesterol, moisture were adjusted

^*^In the subgroup analysis stratified by each covariate, the model is not adjusted for the stratification variable itself. PIR: Ratio of family income to poverty.

## Discussion

The “obesity paradox” has attracted the interest of researchers in the development of many diseases [36–38]. Although chronic diseases of the respiratory system are also affected by the “obesity paradox”, they are mainly lung cancer and chronic obstructive pulmonary disease [39, 40]. The “obesity paradox” is rarely mentioned in the development of asthma because there was a general consensus that obesity could cause a higher prevalence of asthma and the risk of associated adverse events [2]. However, the association between asthma duration and obesity is still not well defined. In the present study, we first demonstrated that an increase with the duration of asthma caused a change in obesity-related indexes. Surprisingly, the correlation between asthma duration and them showed different trends when BMI and WWI were used as response variables, respectively. Because all participants in this study were adults, our results indicated a decrease in weight but an increase in the risk of central obesity with increasing asthma duration in these participants. In adults, BMI basically only responds to changes in body weight. The ability of weight change alone to reflect the true status of fat accumulation and obesity has been questioned by researchers in recent years [21, 22], especially when the concept of muscle-fat-liver axis was introduced in which researchers recognized that weight loss is also very likely to be caused by loss of muscle mass, while visceral fat can be a more true reflection of obesity [41]. Consequently, what we could believe with the present study was that with longer asthma duration, asthmatic patients were at risk of developing more central obesity, even if they were losing weight. In spite of the unadjusted model showing a negative association between asthma duration and WWI, such a finding was validated by trend tests in the full adjusted model after adjusting for all covariates. And the seemingly contradictory correlation between asthma duration and the two different obesity-related indexes in this study might be closely related to muscle steatosis [42], disease-induced reduction in exercise [43], and glucocorticoid use [44], among other reasons. Moreover, we also found that the association between asthma duration and BMI was not always negative, and presented a positive correlation between the two in participants with asthma duration not exceeding 3 years. It is known that muscle has a higher density compared to fat [45], and this phenomenon might be due to the fact that the rate of fat accumulation is greater than the rate of muscle loss early in the onset of asthma, while the disadvantages of muscle loss gradually manifest themselves as the duration of asthma increases. However, there is a positive association between asthma duration and WWI from the beginning to the end, which also indicated that WWI could better reflect the real situation of fat accumulation.

In a subgroup analysis, we found that the association between asthma duration and the two obesity indexes in this study was more significant in participants with early-onset asthma. Although there was no significant difference between early-onset and late-onset asthma in terms of asthma severity [46–48], it was undeniable that early-onset asthma might have a higher risk of steroid use. All of these factors mentioned above are strongly associated with a reduction in muscle mass and the development of central obesity. For example, it has been shown that airway obstruction symptoms were positively associated with decreased muscle mass and activity levels [49], and it has been accepted by all researchers that one of the side effects of glucocorticoids is the development of central obesity [24]. In addition, we found that although female had higher BMI and WWI (Tables 1, 2), the positive correlation between asthma duration and WWI was more pronounced in male participants, which might be related to the fact that the prolonged duration of asthma altered the changes in testosterone in male participants and thus affected the metabolism of visceral fat [50].

As far as we know, this was the first cross-sectional study to explore the relationship between asthma duration and obesity-related indexes in a large sample. However, our study still had several limitations. First, cross-sectional studies cannot explain causality, and follow-up prospective studies are necessary. Second, previous studies have shown that obese patients tend to have worse pulmonary function [27], which may lead to misdiagnosis of asthma in a proportion of obese patients who have not perfected the relevant tests. Therefore, since the diagnosis of asthma in this study was determined by a patient self-reported questionnaire, this also implied that some participants might have been misdiagnosed with asthma. However, based on the current sample size, we concluded that this situation would not ultimately lead to a substantial change in our current results. In addition, the potential influences of asthma duration and obesity-related indices are numerous, and although we included relevant covariates in the models for adjustment based on previous studies, there was no guarantee of bias from other potential covariates. Finally, this was a study based on a cohort of US adults, and the applicability of the current results to populations of other age groups and countries requires follow-up studies.

## Conclusions

There was a negative association between asthma duration and BMI, but a positive association with WWI. Males with asthma onset younger than 12 years of age and aged less than 40 years should be more cautious about the risk of higher WWI. Also, females with asthma onset less than 12 years of age and older than 60 years of age should be aware of a higher risk of weight loss.

## Data Availability

The datasets used and/or analysed during the current study available from the corresponding author on reasonable request.

## Abbreviations

BMI: Body mass index
WWI: Weight-adjusted-waist index
NHANES: National Health and Nutrition Examination Survey
NCHS: National Center for Health Statistics
MECs: Mobile examination centers
PIR: Ratio of family income to poverty
LLR: Log-likelihood ratio
95%CI: 95% Confidence interval
